# Correction: Tan et al. A Systematic Review of the Effects of Caffeine on Basketball Performance Outcomes. *Biology* 2022, *11*, 17

**DOI:** 10.3390/biology11030444

**Published:** 2022-03-15

**Authors:** Zhi Sen Tan, Alexiaa Sim, Masato Kawabata, Stephen F. Burns

**Affiliations:** Physical Education and Sports Science, National Institute of Education, Nanyang Technological University, Singapore 637616, Singapore; tanz0113@e.ntu.edu.sg (Z.S.T.); nie184704@e.ntu.edu.sg (A.S.); masato.kawabata@nie.edu.sg (M.K.)

The authors wish to make a correction to the published version of the paper [1].

In the original publication, the funder “Office of Education Research, National Institute of Education, Nanyang Technological University, Singapore, grant number PG 01/21 SFB, to Stephen F. Burns” was not included. The funder “NTU Research Student Scholarship, National Institute of Education, Nanyang Technological University, Singapore, to Zhi Sen Tan” was also not included.

The authors apologize for any inconvenience caused and state that the scientific conclusions are unaffected. The original publication has also been updated.

The paper should be as follows:

**Funding:** This research was funded by a Planning Grant from the Office of Education Research, National Institute of Education (NIE), Nanyang Technological University, Singapore, grant number PG 01/21 SFB. The first author (Z.S.T.) was supported by an NTU Research Student Scholarship at the National Institute of Education, Nanyang Technological University, Singapore.
